# High density mapping and haplotype analysis of the major stem-solidness locus *SSt1* in durum and common wheat

**DOI:** 10.1371/journal.pone.0175285

**Published:** 2017-04-11

**Authors:** Kirby T. Nilsen, Amidou N’Diaye, P. R. MacLachlan, John M. Clarke, Yuefeng Ruan, Richard D. Cuthbert, Ron E. Knox, Krystalee Wiebe, Aron T. Cory, Sean Walkowiak, Brian L. Beres, Robert J. Graf, Fran R. Clarke, Andrew G. Sharpe, Assaf Distelfeld, Curtis J. Pozniak

**Affiliations:** 1 Department of Plant Sciences, University of Saskatchewan, 51 Campus Drive, Saskatoon, SK, Canada; 2 Swift Current Research and Development Centre, Agriculture and Agri-Food Canada, Swift Current, SK, Canada; 3 Lethbridge Research and Development Centre, Agriculture and Agri-Food Canada, Lethbridge, AB, Canada; 4 Global Institute of Food Security, University of Saskatchewan, 110 Gymnasium Place Saskatoon, SK, Canada; 5 Department of Molecular Biology and Ecology of Plants, Tel Aviv University, Tel Aviv, Israel; Institute of Genetics and Developmental Biology Chinese Academy of Sciences, CHINA

## Abstract

Breeding for solid-stemmed durum *(Triticum turgidum* L. var *durum*) and common wheat (*Triticum aestivum* L.) cultivars is one strategy to minimize yield losses caused by the wheat stem sawfly (*Cephus cinctus* Norton). Major stem-solidness QTL have been localized to the long arm of chromosome 3B in both wheat species, but it is unclear if these QTL span a common genetic interval. In this study, we have improved the resolution of the QTL on chromosome 3B in a durum (Kofa/W9262-260D3) and common wheat (Lillian/Vesper) mapping population. Coincident QTL (LOD = 94–127, *R*^*2*^ = 78–92%) were localized near the telomere of chromosome 3BL in both mapping populations, which we designate *SSt1*. We further examined the *SSt1* interval by using available consensus maps for durum and common wheat and compared genetic to physical intervals by anchoring markers to the current version of the wild emmer wheat (WEW) reference sequence. These results suggest that the *SSt1* interval spans a physical distance of 1.6 Mb in WEW (positions 833.4–835.0 Mb). In addition, minor QTL were identified on chromosomes 2A, 2D, 4A, and 5A that were found to synergistically enhance expression of *SSt1* to increase stem-solidness. These results suggest that developing new wheat cultivars with improved stem-solidness is possible by combining *SSt1* with favorable alleles at minor loci within both wheat species.

## Introduction

The wheat stem sawfly (WSS) (*Cephus cinctus* Norton) is a destructive insect pest of durum (*Triticum turgidum* L var *durum*) and common wheat (*Triticum aestivum* L.) in the northern Great Plains of North America. In Canada, severe infestations of WSS have been reported in southern Alberta, Saskatchewan and eastern Manitoba since the early 1920s [1]. In the United States, areas most prone to sawfly damage include north and eastern Montana, North Dakota, northern South Dakota and western Minnesota [2]. Severe damage has recently been observed in areas of Colorado, Wyoming and Nebraska.

The biology of the WSS has been extensively reviewed [2, 3]. Briefly, WSS emerge from infested stubble of the previous cropping season, usually from around mid-June to mid-July. After mating, the female will select a suitable host plant to puncture using a specialized saw-like ovipositor to deposit an egg. Within five to seven days, the egg will hatch and the process of larval tunneling and feeding on plant tissue within the culm of the stem commences [4]. Larval feeding damages vascular bundles and reduces photosynthetic ability [5]. Kernels harvested from infested plants have 5 to 30% lower mass, and are often of reduced grade [3]. As the wheat host ripens, larvae move towards the base of the plant where they will chew a notch to girdle the stem, fill that region with frass and encase themselves in a hibernaculem to prepare for overwintering. The stem then easily topples over from wind and lodged plants are often not picked up at harvest, causing additional yield losses [6]. A range of agronomic factors have been explored to reduce yield losses by WSS, such as insecticides, tillage, varietal blends, and altered sowing densities [7–11]. An integrated pest management approach centered around growing resistant solid-stemmed cultivars with increased pith in the stem is an effective management approach for WSS.

Growing solid-stemmed wheat cultivars that develop pith in the culm lumen has been the primary strategy to minimize yield losses [2]. Pith increases egg mortality through mechanical crushing [12], and acts as a physical barrier restricting larval movement inside the stem to within one or two internodes from the point of egg deposition [13]. Consequently, WSS survivorship and yield losses are reduced in solid-stemmed cultivars [14]. The expression of stem-solidness can vary between and within common wheat and durum wheat. This may be explained by genetic differences between germplasm sources from which stem-solidness was derived, differences in ploidy between the two species, or other genetic factors.

Research on solid-stemmed wheat has primarily focused on common wheat. Most common wheat cultivars in North America derive their stem-solidness from the Portuguese landrace S-615. The underlying genetics of stem-solidness in the S-615 source are complex, and may include the action of a major gene coupled with four or more additional recessive genes [15]. Many of the S-615 derived cultivars suffer from inconsistent pith expression, because of genetic suppression effects in some wheat backgrounds [16]. In addition, environmental factors such as reduced light intensity during stem elongation, can negatively influence pith development [17]. A number of genetic mapping studies have localized genetic factors contributing to pith development to at least seven chromosomes in common wheat. In S-615, genes influencing stem-solidness were localized to chromosomes 3B, 3D, 5A, 5B, and 5D [16]. The major QTL *Qss*.*msub-3BL* has been shown to explain at least 76% of the variation for stem-solidness in a winter wheat mapping population, and may contain multiple alleles conferring varying levels of stem solidness [18]. A second minor QTL conferring stem-solidness, *Qss*.*msub-3DL*, was localized to chromosome 3DL in a mapping population derived from the semi-solid by solid cross MTHW9904/Choteau [19]. The strong expression of stem-solidness in Choteau over other cultivars is influenced by presence of both *Qss*.*msub-3BL* and *Qss*.*msub-3DL*. Finally, genome-wide association mapping identified novel minor QTL for stem-solidness on chromosomes 2A, 3A and 5B and 5D [20].

Durum wheat has greater stem-solidness compared to many common wheat cultivars [21]. Currently, the solid-stemmed durum cultivars registered for use in western Canada, CDC Fortitude [22], AAC Raymore [23], AAC Stronghold (unpublished), and AAC Cabri [24] all derive their stem-solidness from the German cultivar Biodur. To date, the only mapping work in durum wheat identified a single locus, which was later renamed solid-stem locus 1 (*SSt1*) [25], that was responsible for conferring stem-solidness in the doubled haploid (DH) population Kofa/W9262-260D3, and recombinant-inbred line (RIL) populations Golden Ball/DT379//STD65 and G9580B-FE1C/AC Navigator [26]. The authors suggested that W9262-260D3 (Kyle*2/Biodur) and Golden Ball carry the same single dominant gene for stem-solidness on chromosome 3B, although they did note differences in polymorphisms for certain markers flanking the locus [26].

Identifying the genetic basis for stem-solidness will provide important insight to maximize phenotypic expression in cultivars grown in WSS prone areas. Although QTL conferring stem-solidness have been identified for both common and durum wheat, it is unclear if the genetic basis is the same in both. In addition, many of the existing genetic maps have poor resolution and use different sets of markers, which make them difficult to compare. In this study, we overcame these challenges by using the wheat 90K array, a standardized genotyping platform with high marker density [27]. This technology allowed us to map the stem-solidness QTL in high resolution for both common and durum wheat, as well as compare genetic maps between common and durum wheat. Furthermore, comparison of markers from the wheat 90K array to the high quality wild emmer wheat (WEW) reference sequence allowed us to construct and compare physical map intervals of QTL for both common and durum wheat. Together, our findings shed light on the genetic basis of stem-solidness for both common and durum wheat. Resources developed from this study are currently being used in the development of new wheat cultivars with improved resistance to WSS.

## Materials and methods

### Plant materials

Two bi-parental DH mapping populations were used in this study, which consisted of either durum or common wheat. The first consisted of 155 durum DH lines derived from the cross Kofa/W9262-260D3. Kofa is a hollow-stemmed cultivar from the United States, and W9262-260D3 is a solid-stemmed cultivar derived from the cross Kyle*2/Biodur [26]. Biodur is a solid-stemmed cultivar of German origin that has been used as the predominant source of stem-solidness for modern Canadian solid-stemmed durum cultivars [28]. The second DH mapping population consisted of 293 lines that were derived from the common wheat cross Lillian/Vesper. Lillian is a solid-stemmed cultivar derived from S-615 and has been widely grown in Western Canada for its WSS resistance, high yield, and grain protein content [29].

To validate the results from the bi-parental mapping of the *SSt1* interval, two diversity panels were used for haplotype analysis that included either durum or common wheat. The durum set consisted of 103 cultivars, while the common wheat set contained 98 cultivars. The wheat cultivars in both diversity panels were primarily from North America, with some selections from around the world (S2A and S2B Table).

### Field experiments

All field plots were sown between May and mid-June with a target sowing density of 250 seeds / m^-2^ with 23.5 cm row spacing. The Kofa/W9262-260D3 mapping population was planted in plots located near Swift Current (SK) in a randomized complete block design in 2000 and an alpha lattice in 2001 and 2002, with two replications in each year. The Lillian/Vesper mapping population was planted in 3 m single rows in un-replicated trials near Swift Current (SK) in 2014 and 2015. In 2015, the Lillian/Vesper mapping population was planted as 1m single rows near Saskatoon (SK) in an alpha lattice design with three replications. In addition, the two diversity panels were grown in field nurseries near Saskatoon (SK) in an alpha lattice design in 2011 and 2012, with two replications in each year. Permission to use field sites located at Saskatoon, and Swift Current, was provided by the University of Saskatchewan, and Agriculture and Agri-Food Canada, respectively.

### Phenotyping and statistical analysis of field experiments

The main stem from five to fifteen plants per plot were rated for stem-solidness at maturity using the rating system (1–5) described previously [30]. Each internode was assigned a stem-solidness rating and averaged to obtain an overall rating per plot. Statistical analysis for replicated field trials was performed using the MIXED procedure of SAS/STAT^®^ v9.4. Site years, replications, and blocks were considered as random effects, whereas genotype (i.e. each line) was considered as a fixed effect. Interactions between genotype and all random effects were set as random in the statistical model. Multi-environment least-square means (LS means) for stem-solidness were estimated for each DH line for subsequent use in QTL mapping. The same statistical models were used to generate LS means for each line in the diversity panels. Means separation was performed using Fisher’s Least Significant Difference (LSD) test with a significance value of *p* < 0.05, implemented through the PDMIX800 SAS macro [31].

### Molecular analysis

Genomic DNA was extracted from fresh leaf tissue for each DH line and for the lines from the diversity panels using a modified CTAB approach [32]. DNA quality was examined on agarose gels and diluted to 50 ng/μL. All lines were genotyped using the wheat 90K array [27]. The durum DH population was also genotyped using PCR based markers developed using primer3 software [33] to flank the *SSt1* locus: EK_02–292495 (F-CCACATCAAGGAAACTCAAACA, R-AGCTATAAGACGATGCAAGGCT) and EK_08–5169 (F-AAGCATGGGATGAGAGGAGATA, R-GCCATAGAGAATGCTCCTGTTC) (K. Nilsen, Unpublished data).

### Linkage and QTL mapping

Genotypic data from the wheat 90K array for each mapping population were filtered against markers showing significant segregation distortion (deviating from the expected 1:1 ratio for DH populations) using a chi-square (χ^2^) test. Markers missing 25% or more of the data were removed from the analysis. Draft maps were generated using the MSTMap software [34] with a *p*-value of 1E^-10^ and a maximum distance between markers of 15.0 cM for grouping SNPs into linkage groups. Maps were refined using the MapDisto v1.7.5 software [35] using a threshold LOD score of 3.0 and a cut off recombination value of 0.35. The best order of markers was estimated using both “AutoCheckInversions” and “AutoRipple” commands in MapDisto and distances between markers were calculated using the Kosambi function [36]. Linkage groups (LGs) were scanned and corrected for double recombinants using MapDisto v1.7.5 [35]. Final LGs were assigned to a chromosome based on the existing high density 90K wheat consensus maps [27, 37].

QTL analysis was performed using Windows QTL Cartographer software. Composite interval mapping (CIM) was implemented with a 1.0 cM walk speed. Cofactor selection was performed using forward and backward regression with a significance level of *p* = 0.1 with a 1 cM window size. QTL significance thresholds were determined by permutation tests (1000 permutations) at a significance level of *p* = 0.05. QTL intervals for haplotype analysis were defined by the entire CIM interval above which the LOD score was greater than the calculated threshold value.

### QTL interaction tests

Two-way QTL interactions with *SSt1* were modeled as fixed effects influencing stem-solidness. The closest 90K probe to each QTL peak was used as a diagnostic marker testing for QTL interaction effects within each mapping population. Carriers were distinguished from non-carriers and the stem-solidness LS means were calculated for each. Data were analyzed using the Mixed procedure of SAS v9.4. Means separation was performed using Fisher’s LSD test with a significance value of *p* < 0.05, implemented through the PDMIX800 SAS macro [31].

### In-silico mapping of 90K probe sequences to the WEW reference

In order to determine the physical position of 90K probes along chromosome 3B, GMAP software [38] was used to align the 90K probe source sequences [27] to the complete WEW reference sequence (A. Distelfeld, personal communication). Filtering criteria was applied such that significant hits were required to obtain a minimum threshold sequence identity and coverage, of 95% and 90%, respectively.

### Map comparison and 3B haplotype analysis

Probes from the 90k wheat array that mapped to the QTL intervals on chromosome 3B for the bi-parental DH populations of durum and common wheat were compared to their respective consensus maps[27, 37]. The physical positions of the 90K probes within these intervals on WEW chromosome 3B were used to compare between genetic, and physical distance. Annotated genes falling within the physical intervals were extracted from the WEW gene annotation’s gene transfer format (GTF) file: TRIDC_WEWseq_PGSB_20160501_HighConf.gtf (A. Distelfeld, personal communication).

Using the inferred QTL position on the consensus maps, we also aligned 90K genotypic data from the two diversity panels and identified haplotype groups containing historical recombination events within the *SSt1* interval. Two-dimensional hierarchical cluster analysis was performed using dendextend package of R. v3.3.1. to simultaneously cluster groups of markers, and cultivars based on genotypic similarity. Data were visualized using the Heatmap function of the ComplexHeatmap package of R v3.2.1.

## Results

### Pith expression differences exist between durum and common wheat

**Fig 1:**
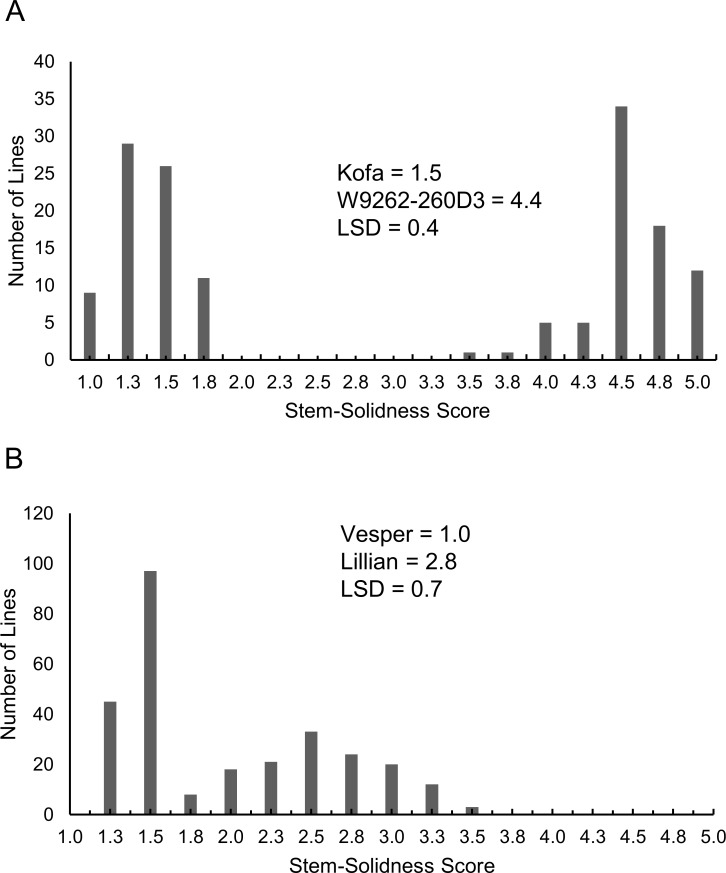
Frequency histograms displaying least-square means for stem-solidness. Scores are averaged across testing environments for DH lines in a) Kofa/W9262-260D3 (durum), and b) Lillian/Vesper (common wheat) mapping populations.

The pattern of phenotypic variation differed for stem-solidness between the two DH mapping populations. The distribution of the stem-solidness phenotype in the Kofa/W9262-260D3 DH population was bimodal with scores ranging from one to five (p < 0.05), with two clear and distinct groups clustering near the extremes of the stem-solidness rating scale (Fig 1A). Some lines exhibited transgressive segregation for stem-solidness, either being more hollow than Kofa (stem-solidness < 1.5), or more solid than W9262-260D3 (stem-solidness > 4.4). The pattern of segregation fit the expected 1:1 expected ratio (*X*^2^ = 0.007, *p* > 0.95) for a single major gene in the DH population, which allowed stem-solidness to be mapped qualitatively as a genetic marker. The pattern of stem-solidness variation in the Lillian/Vesper population ranged from scores of 1.1 to 3.4 (p < 0.05). The distribution of stem-solidness followed an approximate bimodal distribution, but the difference between hollow and solid lines was less pronounced; therefore, discrete classification was not achievable (Fig 1B). The least solid lines were similar to the hollow parent Vesper (stem-solidness = 1.0), whereas the most solid lines exceeded stem-solidness in Lillian, although the difference was not statistically significant (stem-solidness > 2.8).

**Fig 2:**
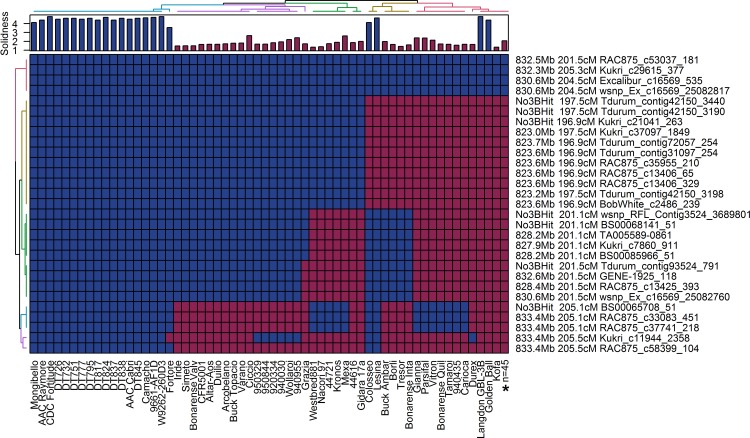
Haplotypes of 103 durum cultivars within the Kofa/W9262-260D3 *SSt1* QTL interval. Stem-solidness LS means for each line are shown in the bar chart along the top X-axis. The matrix consists of 90K genotypic data where cells shaded in blue denote expression of the W9262-260D3 (solid-stem) allele, whereas cells shaded in red denote expression of the Kofa (hollow-stem) allele. The name and position of each 90K probe, the anchored physical position on WEW chromosome 3B, and the corresponding position on the common wheat consensus map are shown. Two dimensional (row and column) hierarchical cluster analysis was performed to group lines into haplotypes as indicated by the colorized dendogram along the top X-axis, whereas similarly marker order is shown along the left Y-axis. *Lines showing identical haplotypes (n = 45) were collapsed into a single haplotype (S2A Table).

Stem-solidness scores from the durum haplotype diversity panel (see Fig 2) ranged from nearly completely solid (stem-solidness = 4.7) to completely hollow (stem-solidness = 1.0). Among the cultivars scoring highest for stem-solidness were the Biodur derivatives: W9262-260D3, CDC Fortitude and AAC Raymore. A high level of stem-solidness was also expressed in Golden Ball, Lesina, Colloseo, Camacho and Fortore. A large proportion of cultivars scored towards the hollow side of the rating scale, which notably included Kofa, the hollow parent of the durum mapping population. A small number of lines expressed intermediate levels of pith (stem-solidness = 2.5–3.5).

**Fig 3:**
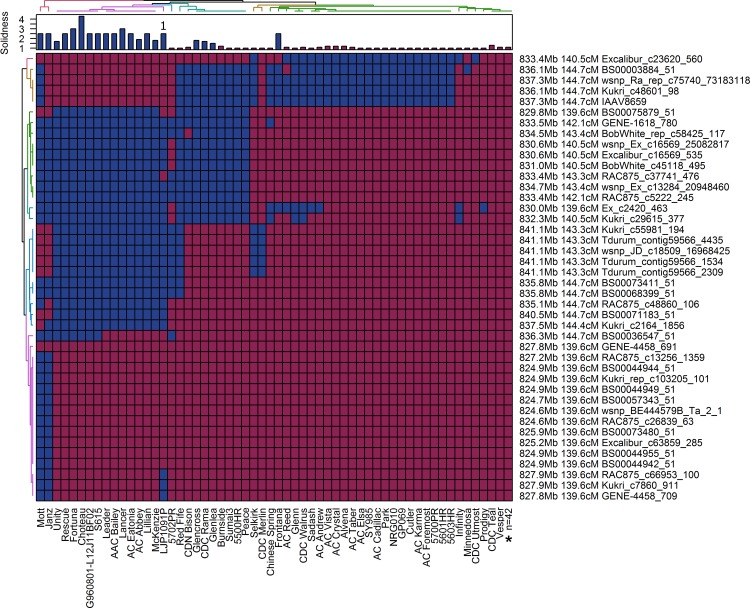
Haplotypes of 98 common cultivars within the Lillian/Vesper *SSt1* QTL interval. Stem-solidness LS means for each line are shown in the bar chart along the top X-axis. The matrix consists of 90K genotypic data where cells shaded in blue denote expression of the Lillian (solid-stem) allele, whereas cells shaded in red denote expression of the Vesper (hollow-stem) allele. The name and position of each 90K probe, the anchored physical position on WEW chromosome 3B, and the corresponding position on the common wheat consensus map are shown. Two dimensional (row and column) hierarchical cluster analysis was performed to group lines into groups as indicated by the colorized dendogram along the top X-axis, whereas markers were grouped along the Y-axis. *Lines showing identical haplotypes (n = 45) were collapsed into a single haplotype (S2B Table). ^1^ Winter wheat, stem-solidness was evaluated on plants grown in a growth chamber.

Stems collected from the common wheat haplotype panel (see Fig 3) ranged from nearly solid to hollow (stem-solidness = 1.0–4.3). The only fully solid-stemmed cultivar was Choteau (stem-solidness = 4.3), whereas the remaining solid cultivars (Lillian, AC Eatonia, AC Abbey, Fortuna, Lancer) had intermediate pith expression (stem-solidness = 2.5–3.5). A number of cultivars expressed small amounts of pith (stem-solidness = 1.5–2), which included McKenzie and Unity, and some members of the Canada Western Extra Strong market class such as Glenlea, CDN Bison and Burnside. Most cultivars in the panel were entirely hollow-stemmed (stem-solidness = 1), which included Vesper, the hollow parent in the common wheat mapping population.

### Stem-solidness is predominantly controlled by the *SSt1* in durum and common wheat

**Fig 4:**
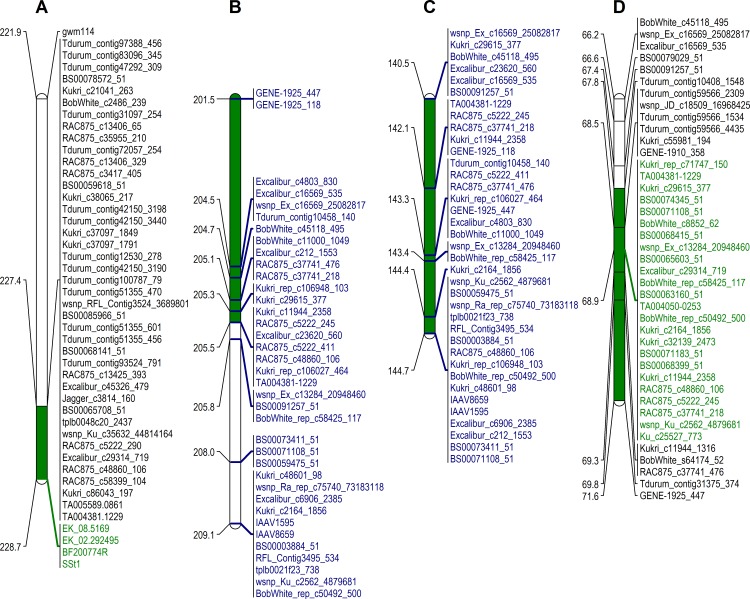
Genetic map interval of *SSt1*. a) Kofa/W9262-260D3 DH population genetic map, b) durum wheat consensus map, c) common wheat consensus map, d) Lillian/Vesper DH population. The position of each QTL is indicated by green shading for each mapping population, and estimated in the consensus map. The markers associated with each QTL peak are highlighted in green text. Common markers between consensus maps are highlighted in blue text.

The wheat 90K array was used to construct a linkage map containing a total of 4227 markers in the Kofa/W9262-260D3 population, which spanned a total map distance of 2282 cM (S1A Table). Stem-solidness in the Kofa/W9262-260D3 population was scored qualitatively (hollow vs. solid) and mapped as a phenotypic marker to position 228.7 cM of chromosome 3B in the genetic map (Fig 4A). Composite interval mapping (CIM) localized significant QTL to chromosomes 3B (*SSt1*), 2A (*Qss*.*usw-2A1*, *Qss*.*usw-2A2*), and 4A (*Qss*.*usw-4A*) (Table 1). The majority of phenotypic variation in this mapping population was explained by *SSt1* (*R*^*2*^ = 92%, LOD = 127), which was localized near the telomere of chromosome 3BL (227.3–228.7 cM, peak = 228.7 cM) (Fig 4A). The peak of the *SSt1* QTL was at position 228.7 cM, which was the same position where *SSt1* was mapped as a phenotypic marker through linkage mapping. The allele conferring stem-solidness at *SSt1* was contributed by the solid parent W9262-260D3. The closest markers to the peak of *SSt1* were PCR-based markers *EK_02–292495*, *EK_08–5169*. The remaining QTL, *Qss*.*usw-2A1*, *Qss*.*usw-2A2* and *Qss*.*usw-4A*, had minor effects with LOD scores ranging from 3.0–5.1, and explained 0.2–0.3% of the phenotypic variance. All three minor QTL had alleles for stem-solidness that were contributed by the hollow parent Kofa. Notably, two distinct QTL were detected on chromosome 2A separated by > 50 cM between the QTL peaks.

**Table 1:** Summary of composite interval mapping (CIM) results. QTL were localized in the Kofa/W9262-260D3 (durum) and Lillian/Vesper (common wheat) mapping populations.

Population	QTL name	CHR	Interval (cM)	Peak position (cM)	LOD	R^2^ (%)	Additive Effect ^1^
Kofa/W9262-260D3	*Qss*.*usw*.*2A*.*1*	2A	81.11–87.1	83.5	3.0	0.2	0.1 (K)
	*Qss*.*usw*.*2A*.*2*	2A	129.7–155.6	137.7	5.1	0.3	0.1 (K)
	*SSt1*	3B	227.3–228.7	228.7	126.9	92.1	1.6 (W)
	*Qss*.*usw*.*4A*	4A	112.2–137.1	125.5	3.0	0.2	0.1 (K)
Lillian/Vesper	*Qss*.*usw*.*2D*	2D	91.3–131.4	112.9	6.1	2.8	0.1 (L)
	*SSt1*	3B	67.8–71.6	68.9	94.0	77.8	0.6 (L)
	*Qss*.*usw*.*5A*	5A	88.5–100.3	92.3	3.9	1.3	0.1 (L)

CHR, Chromosome

LOD, Logarithm of Odds

^1^Parent contributing positive allele, K = Kofa, W = W9262-260D3, L = Lillian, V = Vesper

The Lillian/Vesper genetic map contained 7839 markers, which covered a total map distance of 3680 cM (S1B Table). Significant QTL were localized to chromosomes 3B (*SSt1*), 2D (*Qss*.*usw-2D*), and 5A (*Qss*.*usw-5A*) (Table 1). The majority of the phenotypic variation was explained by *SSt1* (LOD = 94.0, R^2^ = 77.8%), which spanned from map position 67.8–71.6 cM (Peak position = 68.9 cM) (Table 1, Fig 4D). The remaining QTL, although significant, had only minor effects (*R*^*2*^ = 1.3–2.8%). The alleles conferring stem-solidness at all QTL in the Lillian/Vesper cross were contributed by the solid parent Lillian.

### Synergistic QTL interactions enhance the effect of *SSt1*

The major locus *SSt1* on chromosome 3B, has been previously shown to interact epistatically with other minor QTL to synergistically enhance expression of stem-solidness [18]. In the present study, transgressive segregation was observed for stem-solidness in some cases, therefore the possibility of synergistic interaction between QTL was investigated in further detail. These results indicated that the two-way interactions between *SSt1* and all minor QTL were all strongly significant (*p<*0.01) in both mapping populations. In the Kofa/W9262-260D3 population, the combination of alleles conferring stem-solidness in two-way interactions (SSt1*Qss.*usw-2A*.*1*, SSt1*Qss.*usw-2A*.*2*, SSt1*Qss.*usw-4A)* conferred stem-solidness that exceeded the score in W9262-260D3 (stem-solidness > 4.4; Table 2). However, in the absence of *SSt1*, DH lines carrying solidness alleles at each minor QTL did not express significantly more pith than non-carriers. The only exception was lines carrying *Qss*.*usw-2A*.*1*, which had some minor pith development independent of *SSt1* (stem-solidness = 1.4,). In the Lillian/Vesper population, the presence of stem-solidness alleles at each minor QTL acted synergistically with *SSt1* to significantly increase pith density compared to lines carrying only *SSt1* (*p*< 0.05) (Table 2). However, no two-way interaction between *SSt1*, and minor QTL, yielded stem-solidness that exceeded that of Lillian (stem-solidness = 2.8,). In the absence of *SSt1*, none of the minor QTL had a significant effect on pith development in the Lillian/Vesper population.

**Table 2:** Synergistic two-way interactions between *SSt1* and minor QTL identified in the Kofa/W9262-260D3 (durum) and Lillian/Vesper (common wheat) mapping populations.

**Kofa/W9262-260D3**					
***SSt1***^1^	***Qss*.*usw-2A1***	***Qss*.*usw-2A2***	***Qss*.*usw-4A***	**Stem-solidness**^**2**^	**SE**
+	+			4.56^a^	0.04
+	-			4.33^b^	0.04
-	+			1.41^c^	0.05
-	-			1.21^d^	0.04
+		+		4.55^a^	0.04
+		-		4.34^b^	0.04
-		+		1.32^c^	0.05
-		-		1.30^c^	0.04
+			+	4.54^a^	0.04
+			-	4.35^b^	0.04
-			+	1.32^c^	0.04
-			-	1.29^c^	0.04
**Lillian/Vesper**					
***SSt1***	***Qss*.*usw-2D***	***Qss*.*usw-5A***			
+	+			2.51^a^	0.04
+	-			2.32^b^	0.04
-	+			1.34^c^	0.04
-	-			1.28^c^	0.04
+		+		2.54^a^	0.04
+		-		2.29^b^	0.04
-		+		1.34^c^	0.04
-		-		1.29^c^	0.04

^1^‘+’ denotes the group carries the stem-solidness allele for the specified QTL, whereas ‘-’ denotes the group carries the stem-hollowness allele. Blank cells indicate the QTL was not considered for the particular comparison.

^2^LS means for stem-solidness for each two-way allele combination (1–5 scale). Letter groupings in superscript statistical significance between LS means determined through Fishers LSD test at *p*< 0.05.

### Comparison of *SSt1* using wheat consensus maps

In order to facilitate the comparison between the *SSt1* interval in the Kofa/W9262-260D3 and Lillian/Vesper mapping populations, the position of 90K probes within each QTL interval were compared to the published durum and common wheat consensus maps (Fig 4B and 4C). Markers mapping within the *SSt1* interval in Kofa/W9262-260D3 spanned from positions 196.3 to 205.5 cM on the durum consensus map (Fig 4B). The remaining 90K probes distal to position 205.5 cM were not polymorphic between Kofa and W9262-260D3. The markers in the *SSt1* interval in Lillian/Vesper interval spanned 140.5–144.7 cM in the hexaploid consensus map (Fig 4C). Comparison of 90K probes common to the two consensus maps revealed that the two QTL span a similar genetic interval on chromosome 3B, and there were 22 common 90K probes between the two consensus maps (Fig 4B and 4C). Overall, the co-localization of the markers for in the Kofa/W9262-260D3 linkage map were in agreement with their positions in the durum consensus map (Fig 4A and 4B). Similarly, the markers in the Lillian/Vesper linkage map were in agreement with the common wheat consensus map, although some minor differences in marker order were noted (Fig 4C and 4D).

### 90k probes from *SSt1* are coincident in common and durum wheat

The relationship between physical and genetic map positions was assessed by mapping 90K probe sequences against the WEW chromosome 3B reference sequence. Probe sequences that did not meet the minimum sequence identity (>95%) and coverage requirements (> 90%) and were removed from the analysis. The physical location of markers closest to the peak of *SSt1* in Kofa/W9262-260D3 spanned positions 823.0–835.1 megabase pairs (Mb) on WEW chromosome 3B (Fig 2). The order of the probes on chromosome 3B was consistent with their position on the durum consensus map (Fig 2). The estimated position of the 3B QTL in the Lillian/Vesper population spanned positions 140.5–144.7 cM on the common wheat consensus map (Fig 4C). The 90K probes within this interval on chromosome 3B in WEW ranged from positions 830.6–841.0 Mb (Fig 3). Based on the 22 probes in *SSt1* that were shared between the common and durum wheat consensus maps, spanning 140.5–144.7 cM in common wheat and 204.5–209.1 cM. in durum wheat, there is a region of overlap; this region corresponds to positions 830.2–837.5 Mb in WEW chromosome 3B.

### Diversity panels reveal multiple *SSt1* haplotypes

Further investigation of the *SSt1* interval in the durum diversity panel identified six different haplotype groups within the Kofa/W9262-260D3 QTL interval (Fig 2). Most of the solid-stemmed Biodur derivatives, including the CDC Fortitude and AAC Raymore were part of a haplotype group that was identical to W9262-260D3. In addition, Camacho and 9661.AF1D also carried the haplotype identical to Biodur and the solid line Fortore was nearly identical to Biodur, except at the marker *Kukri_c11944_2358* (Fig 2). The solid Italian cultivars Lesina and Colloseo had unique haplotypes, and carried the Kofa allele between WEW chromosome 3B positions 823.0–823.7 Mb, and the W9262-260D3 allele at all remaining loci within the QTL interval. The majority of lines in the panel showed an identical haplotype to Kofa and had hollow stems, with the exception of the solid-stemmed lines Langdon-GB-3B and Golden Ball (Fig 2). The only marker to properly differentiate all solid from hollow lines in the panel (with the exception of Golden Ball, and Langdon-GB-3B) was *RAC875_c58399_104*, which was located at WEW position chromosome 3B 833.4 Mb (consensus 205 cM). Not only was this marker the most distally located marker in the dataset, but it was also the most distal marker in the durum consensus map that was polymorphic between the parents of the durum mapping population Kofa and W9262.

Within the common wheat QTL interval, a total of five different haplotype groups were identified through hierarchical cluster analysis (Fig 3). Most solid-stemmed derivatives of S-615, which included AAC Bailey, Unity, Rescue, Fortuna, Choteau, Leader, Lancer, Mckenzie, and AC Abbey, were nearly identical in haplotype to Lillian and carried the allele for stem-solidness between WEW 3B positions 830.0–841.1 Mb on chromosome 3B. A second haplotype was identified consisting of several members from the Canada Western Extra Strong (CWES) market class which carried the stem-solidness allele between 830.0–837.3 cM. This group also contained the hollow-stemmed lines Sumai 3, Peace, 5500HR, and Red Fife (Fig 3). The pattern of pith expression within this group was split between the CWES cultivars, which had low to intermediate pith development, and the other lines Sumai 3, Peace, 5500HR, and Red Fife which were entirely hollow-stemmed. Finally, there was a unique haplotype that consisted of the solid-stemmed lines Mott and Janz. The majority of lines in the panel consisted of hollow-stemmed cultivars, which had shared identical haplotype to Vesper (Fig 3).

### Candidate genes contributing to stem-solidness in *SSt1*

Based on the peak of *SSt1* in common wheat (832.2–835.1 Mb) and durum wheat (833.5–833.6 Mb) (Table 1), and overlapping 90k probes and haplotypes for common and durum wheat (Fig 2, Fig 3), we were able to narrow the genetic interval for *SSt1* to 833.4–835.0 Mb in WEW chromosome 3B. This interval contains 43 high confidence genes, based on the current version of the WEW annotation. Of these, 23 are classified as having unknown function (Table 3). Of the 20 functionally annotated genes, notable candidates for the solid-stem phenotype include three ribosomal proteins (RPS17, RPS19 and RPS28), a Dof zinc finger transcription factor (Dof2), and a protein kinase superfamily protein (Table 3).

**Table 3:** High confidence annotated genes within the *SSt1* interval in WEW chromosome 3B.

Gene ID	Description	Emmer Start	Emmer End
TRIDC3BG086390	AP-3 complex subunit beta-2	833,410,411	833,417,858
TRIDC3BG086400	unknown function	833,418,228	833,419,311
TRIDC3BG086410	unknown function	833,447,122	833,448,100
TRIDC3BG086420	Dual-specificity RNA methyltransferase RlmN	833,471,636	833,473,995
TRIDC3BG086430	Protein kinase superfamily protein	833,499,650	833,502,363
TRIDC3BG086440	undescribed protein	833,500,052	833,501,188
TRIDC3BG086450	undescribed protein	833,568,672	833,569,127
TRIDC3BG086460	40S ribosomal protein S28	833,617,967	833,619,969
TRIDC3BG086470	undescribed protein	833,695,443	833,696,292
TRIDC3BG086480	unknown function	833,753,129	833,755,302
TRIDC3BG086490	unknown function	833,960,480	833,981,832
TRIDC3BG086500	undescribed protein	834,115,057	834,115,259
TRIDC3BG086510	Protein of unknown function (DUF506)	834,115,507	834,117,890
TRIDC3BG086520	undescribed protein	834,115,959	834,116,369
TRIDC3BG086530	NAD(P)H-quinone oxidoreductase subunit 6, chloroplastic	834,154,291	834,154,828
TRIDC3BG086540	undescribed protein	834,278,312	834,279,220
TRIDC3BG086550	undescribed protein	834,278,400	834,278,856
TRIDC3BG086560	Vacuolar protein sorting-associated protein 25	834,313,166	834,344,089
TRIDC3BG086570	Disease resistance protein RPM1	834,329,315	834,331,881
TRIDC3BG086580	12S seed storage globulin 2	834,354,104	834,355,541
TRIDC3BG086590	Accelerated cell death 11	834,398,396	834,398,890
TRIDC3BG086600	undescribed protein	834,399,338	834,400,223
TRIDC3BG086610	12S seed storage globulin 1	834,443,909	834,445,752
TRIDC3BG086620	undescribed protein	834,473,716	834,474,056
TRIDC3BG086630	undescribed protein	834,501,444	834,501,784
TRIDC3BG086640	undescribed protein	834,529,195	834,530,259
TRIDC3BG086650	30S ribosomal protein S17	834,546,892	834,549,240
TRIDC3BG086660	30S ribosomal protein S19	834,559,426	834,561,567
TRIDC3BG086670	unknown function	834,649,174	834,650,437
TRIDC3BG086680	Mitochondrial ATP synthase 6 kDa subunit	834,677,496	834,677,663
TRIDC3BG086690	undescribed protein	834,687,517	834,688,148
TRIDC3BG086700	undescribed protein	834,688,939	834,691,009
TRIDC3BG086710	Very-long-chain (3R)-3-hydroxyacyl-CoA dehydratase 2	834,691,153	834,693,605
TRIDC3BG086720	DOF zinc finger protein 2	834,983,287	834,984,049
TRIDC3BG086730	undescribed protein	835,036,191	835,036,444
TRIDC3BG086740	undescribed protein	835,037,520	835,037,765
TRIDC3BG086750	undescribed protein	835,075,268	835,075,570
TRIDC3BG086780	Transposon protein, putative, CACTA, En/Spm sub-class	835,127,161	835,133,343
TRIDC3BG086800	Transposon protein, putative, CACTA, En/Spm sub-class	835,129,795	835,130,231
TRIDC3BG086810	Ankyrin repeat family protein	835,176,160	835,177,639
TRIDC3BG086820	undescribed protein	835,177,635	835,178,250
TRIDC3BG086830	Cytochrome P450 superfamily protein	835,354,094	835,355,636
TRIDC3BG086840	unknown function	835,360,730	835,361,289

## Discussion

In this study, we localized coincident QTL conferring stem-solidness to chromosome 3BL in the durum population Kofa/W9262-260D3, and common wheat population Lillian/Vesper. The QTL interval on chromosome 3B in durum wheat was consistent with the previously reported location of *SSt1* [26]; similarly, the QTL interval in Lillian/Vesper was consistent with the previously reported location of *Qss*.*msub-3BL* [18]. Earlier work identified two 90K probes (*BS00065603* and *BS00074345_51*) in linkage disequilibrium (LD) with *Qss*.*msub-3BL* through association mapping [20]. In the present study, both markers co-segregated with the peak of the Lillian/Vesper QTL (Fig 4D), indicating the Lillian/Vesper QTL is indeed coincident with *Qss*.*msub-3BL*. Comparison of the wheat consensus maps identified 22 common probes within the QTL intervals in durum and common wheat (Fig 4B and 4C), with probe sequences that spanned a physical interval of 830.2–837.5 Mb. Based on the peaks of the QTL, overlapping marker, and haplotype evidence, we have further defined this interval to approximately 2 Mb (833.4–835.1 Mb). Since these QTL are coincident in their physical and genetic maps, we suggest that they correspond to the same region in both wheat species, which we henceforth designate *SSt1*. If common and durum wheat carry a common gene within *SSt1* that confers stem-solidness on chromosome 3B, then it may be localized to this common physical interval between the two defined QTL.

Within the common interval in WEW, there are 43 putative high confidence annotated genes, several of which could be involved in biological processes related to stem-solidness. These include three ribosomal proteins (RPS17, RPS19, RPS28), a Dof zinc finger transcription factor (Dof2), and a protein kinase superfamily protein. Increased expression of ribosomal proteins (RPs) would be expected in actively dividing tissues including the pith of solid-stemmed cultivars. There are 70–80 different types of RPs and are required to be in stoichiometric balance to make up the ribosomal complex responsible for protein synthesis [39]. Defects in part of the ribosomal protein complex can result in cell-cycle arrest via apoptosis in animal systems [40]. Therefore, it could be possible that mutations affecting the function of a specific ribosomal protein could cause the hollow-stemmed phenotype. On the other hand, Dof proteins (DNA-binding with one finger) are a family of transcription factors specific to plants responsible positive and negative regulation of gene expression implicated in a wide variety of functions, including cell cycle regulation [41], cell cycle progression/cell expansion [42], photosynthesis and light response, and plant growth and plant development [43]. Likewise, protein kinases are involved in post translational modification of proteins and signal transduction, and similar to the Dof transcription factor could be involved in a wide array of processes [44]. Work is currently underway to determine whether these, or other genes, are differentially expressed in the pith of developing plants and if they contain genetic variants between hollow and solid-stemmed parents that could explain the differential phenotypic response.

In addition to *SSt1*, we also observed that synergistic two-way interactions between *SSt1* and other minor QTL on chromosomes conferred a greater level of stem-solidness than the presence of *SSt1* alone. We identified minor QTL on chromosomes 2A, and 4A in our durum mapping population (Kofa/W9262-260D3), and 2D, and 5A in the common wheat population (Lillian/Vesper). Previous studies have also identified minor QTL conferring stem-solidness. For example, a secondary QTL was identified on chromosome 3DL that enhances pith expression when combined with *SSt1*[19]. In the present study, the solid-stem alleles for the durum QTL on 2A and 4A were contributed by the hollow parent Kofa, which suggests that some hollow by solid parental combinations could be used to enhance expression of stem-solidness in durum wheat. This may not be of critical importance to durum wheat breeders because modern cultivars that carry *SSt1* have strong pith expression that exceeds the minimum threshold stem-solidness score of 3.75 proposed to achieve effective sawfly resistance [45]. In the present study, we observed that the additive effect of the *SSt1* resistance allele in durum wheat conferred three times more units of stem-solidness than it did in common wheat. In contrast, variable pith expression has often been an issue for many common wheat cultivars [46]. The variability in common wheat can be caused by environmental conditions, particularly low light intensity during stem elongation, which can negatively impact pith development [17]. Some common wheat cultivars have been shown to express greater amounts of pith at early stages of development when WSS infestation typically occurs, followed by rapid pith retraction towards maturity [47]. In some common wheat cultivars, the presence of *SSt1* alone may not be enough to ensure effective WSS resistance, therefore developing common wheat cultivars with improved WSS resistance remains a priority in breeding programs. We have shown here that some two-way combinations between *SSt1* and minor QTL in the Lillian/Vesper population resulted in stem-solidness that exceeded the effects of *SSt1* alone. These results indicate that future work should include attempts to pyramid *SSt1* with one or more secondary genes with complementary additive effects. Such favorable interactions likely have already been inadvertently implemented by breeding programs through the selection of elite cultivars with increased stem-solidness.

Several different haplotypes were found within the *SSt1* interval in common and durum wheat. All known solid-stemmed cultivars in the durum haplotype panel, with the exception of Golden Ball and Langdon-GB-3B, carried alleles for stem-solidness somewhere within the *SSt1* interval in the Kofa/W9262-260D3 mapping population. The lack of similarity between Golden Ball and the other solid durum lines was unexpected, because the gene conferring stem-solidness in Golden Ball was mapped to a similar region of 3B in a previous study [26]. The solid-stemmed parent (W9262-260D3) of the durum mapping population derives its stem-solidness from the German cultivar Biodur, as do the four commercially registered Canadian durum cultivars CDC Fortitude [28], AAC Raymore [23], AAC Cabri [24], and AAC Stronghold (Unpublished data), and the majority of solid-stemmed Canadian durum breeding lines. Biodur (Valdur//Wascana/Durtal) may derive the solid allele from North African ancestors; the ancestry of Golden Ball is unknown, being a landrace introduced to North America from South Africa in the early 20^th^ century. Therefore, these results could suggest that Golden Ball and Biodur represent different sources of stem-solidness on chromosome 3B. In the present study, a lack of polymorphic markers between Kofa and W9262-260D3 distal to the expected location of *SSt1* hindered comparison between the two putative sources. Therefore, future investigation will be required to confirm whether the gene in Golden Ball is allelic to Biodur.

Alternate haplotypes were also evident in the solid cultivars of Italian origin. Lesina (Capeiti/Creso//Trinakria/Valforte) and Colosseo (Creso/Mexa) expressed stem-solidness similar to Golden Ball, yet had different haplotypes than either the Biodur derivatives or Golden Ball. Fortore (Capeiti 8/Valforte) had a lower stem-solidness score than the Biodur derivatives, but similar haplotype, except at three loci. Conversely, Mongibello (Trinakria/Valforte) was very solid and had the Biodur haplotype, despite similar ancestry to the other solid Italian lines. Of the ancestral lines of these cultivars, we can only confirm stem-solidness in Trinakria [48], although one could speculate that Creso, a cross between a Capelli short straw mutant and a CIMMYT semidwarf line [48] is also solid-stemmed. Together, evidence indicates that Italian cultivars have solid-stem phenotypes, though they do not fully fall within either the Golden Ball or Biodur haplotypes.

Several notable haplotypes were also identified in the common wheat haplotype panel. Many North American common wheat cultivars studied, including Lillian, Rescue, AC Eatonia, AC Abbey, Leader, Lancer, McKenzie and Unity, derive their stem-solidness from the Portuguese landrace S-615 [25, 49]. In the present study, most of the S-615 derivatives carried an identical haplotype to Lillian throughout the QTL interval. However, a distinct haplotype was identified in the solid-stemmed cultivars Mott and Janz. Mott is a spring wheat cultivar developed at North Dakota State University, with stem-solidness that is partially derived from S-615 via the cultivars Ernest, Fortuna and Tioga. In contrast, Janz is a white spring wheat that derives its stem-solidness from an alternative source, *Agropyron elongatum* [49]. Another interesting haplotype was identified in members of the Canada Western Extra Strong (CWES) market class (Glenlea, RL4452, Burnside, Glencross, CDN Bison, CDC Rama) which carry alleles for both solid and hollow stem within the QTL interval. Although this haplotype group consists of cultivars that were relatively hollow-stemmed, certain cultivars such as Glenlea, and CDC Rama did express some pith in the lower internodes, which could indicate they are carriers of *SSt1* with phenotypic suppression of stem-solidness. A number of genes inhibiting the expression of stem-solidness have been identified in S-615 and its derivatives, including those carried by the D-genome [50, 51].

## Conclusions

In conclusion, the major QTL on chromosome 3BL identified in this study is coincident with the previously reported map positions of *Qss*.*msub-3BL* [18] and *SSt1* [26]. To elucidate the relationship between the genetic and physical maps of *SSt1*, we anchored 90K probes that mapped inside the QTL interval to the WEW reference sequence. Combined with haplotype analysis, the most probable location of *SSt1* is estimated to be between positions 833.4–835.1 Mb. The two sources of stem-solidness in durum wheat (Golden Ball and Biodur) are different in haplotype around *SSt1* although QTL have been mapped to 3B in both sources [26]. Golden Ball carries the hollow allele throughout the entire durum *SSt1* interval which will require further investigation to confirm whether it is allelic to *SSt1*. Common wheat cultivars that derived their stem-solidness from S-615 were similar in haplotype, though alternate haplotypes were identified. Despite sharing a common locus on chromosome 3B, phenotypic expression of stem-solidness differed between durum and common wheat. Minor QTL were shown to synergistically enhance the expression of *SSt1* in both mapping populations, which suggests breeding efforts can improve pith expression through strategic parental selection, which may be particularly useful in breeding common wheat.

## Supporting information

S1 Table High density genetic linkage maps. **(**S1A) Kofa/W9262-260D3. (S1B) Lillian x Vesper populations.
(XLSX)

S2 Table Diversity panel haplotypes. (S2A) Tetraploid wheat panel. (S2B) Common wheat panel.
(XLSX)
